# Comparing the impact of an icon array versus a bar graph on preference and understanding of risk information: Results from an online, randomized study

**DOI:** 10.1371/journal.pone.0253644

**Published:** 2021-07-23

**Authors:** Peter Scalia, Danielle C. Schubbe, Emily S. Lu, Marie-Anne Durand, Jorge Frascara, Guillermina Noel, A. James O’Malley, Glyn Elwyn

**Affiliations:** 1 The Dartmouth Institute for Health Policy and Clinical Practice, Geisel School of Medicine at Dartmouth, Lebanon, New Hampshire, United States of America; 2 UMR 1095, Université de Toulouse, Inserm, Université Toulouse III Paul Sabatier, Toulouse, France; 3 Unisanté, Centre Universitaire de Médecine Générale et Santé Publique, Lausanne, Switzerland; 4 Department of Art and Design, University of Alberta, Edmonton, Alberta, Canada; 5 Lucerne School of Arts and Design, Lucerne University of Applied Sciences and Arts, Luzern-Emmenbrucke, Switzerland; 6 Department of Biomedical Data Science, Geisel School of Medicine at Dartmouth, Lebanon, New Hampshire, United States of America; University College London, UNITED KINGDOM

## Abstract

**Background:**

Few studies have examined the best way to convey the probability of serious events occurring in the future (i.e., risk of stroke or death) to persons with low numeracy or graph literacy proficiency. To address this gap, we developed and user-tested a bar graph and compared it to icon arrays to assess its impact on understanding and preference for viewing risk information.

**Objectives:**

To determine the: (i) formats’ impact on participants’ understanding of risk information; (ii) formats’ impact on understanding and format preference across numeracy and graph literacy subgroups; (iii) rationale supporting participants’ preference for each graphical display format.

**Methods:**

An online sample (evenly made up of participants with high and low objective numeracy and graph literacy) was randomized to view either the icon array or the bar graph. Each format conveyed the risk of major stroke and death five years after choosing surgery, a stent, or medication to treat carotid artery stenosis. Participants answered questions to assess their understanding of the risk information. Lastly, both formats were presented in parallel, and participants were asked to identify their preferred format to view risk information and explain their preference.

**Results:**

Of the 407 participants, 197 were assigned the icon array and 210 the bar graph. Understanding of risk information and format preference did not differ significantly between the two trial arms, irrespective of numeracy and graph literacy proficiency. High numeracy and graph literacy proficiency was associated with high understanding (p<0.01) and a preference for the bar graph (p = 0.01).

**Conclusion:**

We found no evidence to demonstrate the superiority of one format over another on understanding. The majority of participants preferred viewing the risk information using the bar graph format.

## Introduction

Understanding the probability of a serious event (i.e., stroke or death) occurring over different time horizons impacts patients’ treatment decisions. Many graphical display formats have been designed to help patients understand the changes in risk over time. However, not many formats have been tested with patients who have lower numeracy and graph literacy skills. Insufficient evidence exists to confidently endorse one particular graphical display format to improve understanding of risk information, particularly among patients with lower numeracy and graph literacy skills [1].

Graphical display formats, some of which include icon arrays, bar graphs, and line or pie charts, are associated with improving risk perception, understanding of numerical data, and increasing the accuracy and speed at which individuals process information [2–7]. A body of evidence supports the use of icon arrays (typically presented as 100 icons separated by color) to help individuals effectively interpret risk information and help reduce cognitive biases like base-rate neglect [6–11]. In terms of improving understanding, there are mixed results across studies that evaluate which graphical display format is superior [12–18]. Some studies indicate that icon arrays help people distinguish whether one quantity is larger than another and to estimate whether there is a difference between two quantities [4, 6, 7]. Most of those studies have been done with people who have higher levels of graph literacy and numeracy [11, 14, 18]. Other studies show that bar charts are just as effective as icon arrays [17, 19], and may have advantages for people who have lower levels of graph literacy and numeracy [20]. However, in general, research aiming to evaluate the effects of graphical display formats on understanding of risk information have typically not included individuals with low levels of objective numeracy and graph literacy [21].

Objective numeracy can be defined as the ‘ability to understand numbers or percentages’ [22]. Fagerlin et al. report that approximately 22% of Americans score in the lowest performance level for objective numeracy which means that they can only solve single-operation math problems [23]. Furthermore, survey research indicates that only 9% of Americans score in the highest numeracy proficiency level [24]. With regards to graph literacy, the ‘ability to understand graphically-presented information’ [25], an estimated 33% of American adults had low graph literacy based on their difficulty understanding standard visual displays [3]. Based on this data, a gap exists in the risk communication literature which contains very few studies on how best to present risk information to persons with lower numeracy and lower graph literacy skills [26].

In an attempt to address this gap, we recruited a sample of participants with high and low numeracy and graph literacy skills to determine the differential impact of two graphical display formats—an icon array versus a bar graph—on their understanding of risk information. Each format conveyed the risk of major stroke and death five years after choosing surgery, a stent, or medication to treat carotid artery stenosis. We also sought to determine participants preferred graphical display format (icon array versus bar graph) to view the risk information.

We aimed to: (1) evaluate the impact of the graphical display formats on participants’ understanding of the risk information presented; (2) examine whether or not preference and understanding are related to high and low objective numeracy and graph literacy proficiency; (3) determine the rationale supporting participants’ preference for each graphical display format.

## Methods

### Design

**Fig 1:**
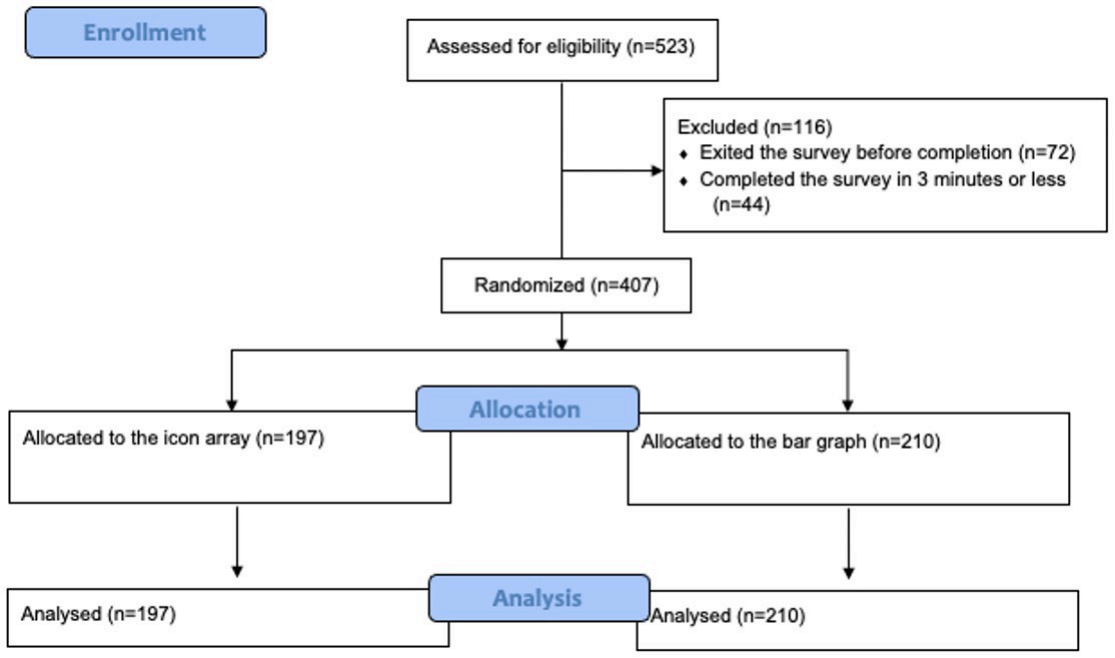
Flow diagram of the study design.

We conducted an online randomized trial in which participants with varying objective numeracy and graph literacy proficiency were randomly assigned to view an icon array or a bar graph. Each format conveys the risk of stroke and death for three carotid artery stenosis treatment options five years post-intervention (see Fig 1 for the flow diagram of our study design). Given the randomization methods used, we adapted the CONSORT reporting guidelines (see S1 Table for details) [27]. Ethical approval for our study was received from the Dartmouth College Committee for the Protection of Human Subjects (STUDY00032004).

### Recruitment

To recruit participants, we contracted Qualtrics, a survey technology company, to provide access to panels of people willing to respond to online surveys [28]. Qualtrics invited potential participants to complete a research survey by email. The email included the survey URL, information on the length of the survey, and a description of the incentive for completing the survey. For legal purposes, Qualtrics did not provide us with any details on the incentives for participants for completing the survey, except that the incentive is ‘based on the length of the survey, the panelist profile, and target acquisition difficulty, amongst other factors’ [28]. The survey invitation did not include details about the research study, although this information appeared in the consent page when the participant clicked the survey URL. Qualtrics ensured the validity of the results by using digital fingerprinting technology and IP address checks and cookies to prevent participants from completing the same survey multiple times. We provided Qualtrics with our sample size, survey length, and screening criteria (see procedure section).

### Participants

Qualtrics included participants who were at least 18 years of age and fluent in the English language. Participants were excluded if they left the survey before completion. Qualtrics estimated the survey time completion to be six minutes, so we agreed with their recommendation to exclude those who completed the survey in three minutes or less (‘speeders’). Qualtrics did not suggest or advise putting a limit on the amount of time needed to complete the survey, so we did not impose a time cap on the survey. We did not exclude participants based on gender, socioeconomic status, educational attainment or any other criteria.

### Interventions

We compared two graphical display formats: the icon array and the bar graph. Each format displays the risk of major stroke and death five years after choosing surgery, a stent, or medication to treat blockage in the carotid artery. We chose the 5-year timeframe based on evidence that shorter timeframes (<10 years) lead to more accurate perceptions of risk [29]. The risk data presented in each format is identical and was derived from the Vascular Quality Initiative (VQI)–a national collaborative that includes patients from 45 states across the US who have undergone vascular surgery [30]. We presented data for the carotid artery stenosis condition as we had already calculated the risks for a previous qualitative study which assessed format preference for conveying the risk of stroke and death to patients [31].

#### Icon array design

**Fig 2:**
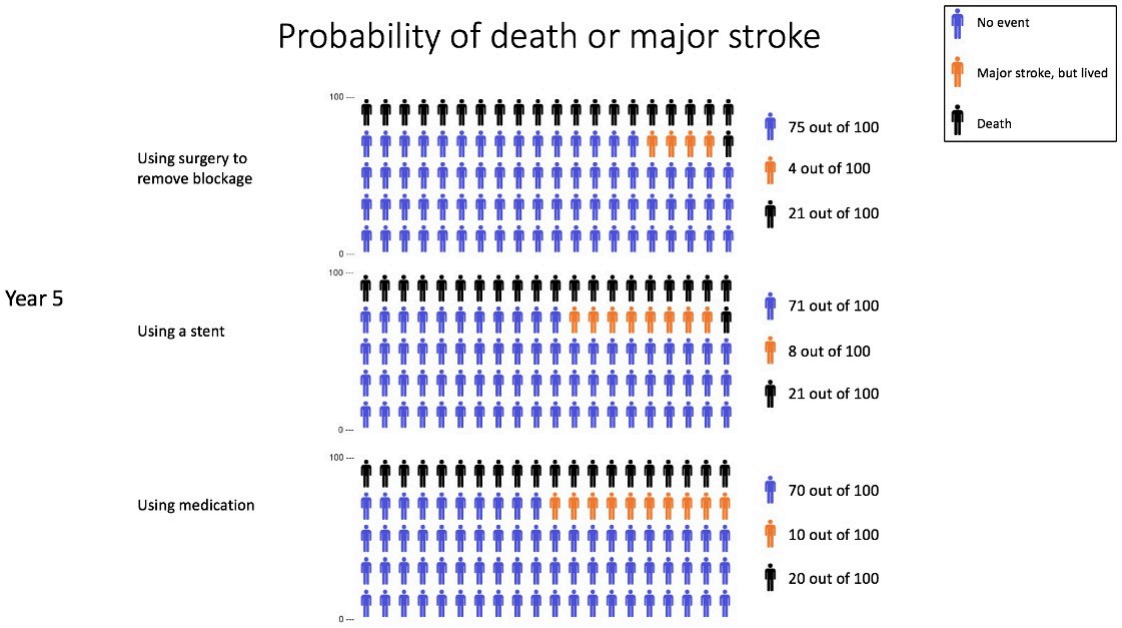
Icon array presented in the survey.

The method used to develop the icon array employed in this study is described elsewhere [31]. In summary, three iterations of qualitative interviews with patients who had carotid artery occlusion informed the treatment labels, colors and layout of the risk information. We created one icon array for each treatment option (see Fig 2). Using a denominator of 100 icons and a legend, we used blue icons to represent the chance that ‘no one suffered a stroke or died’, orange icons represented the chance of ‘major stroke, but lived’, and black icons represented the chance of ‘death’. We presented the probabilities of major stroke and death beside each icon array as natural frequencies (i.e., 75 out of 100) as recommended in the literature, to reduce misinterpretation and facilitate risk understanding [5, 10, 32–34].

#### Bar graph design

**Fig 3:**
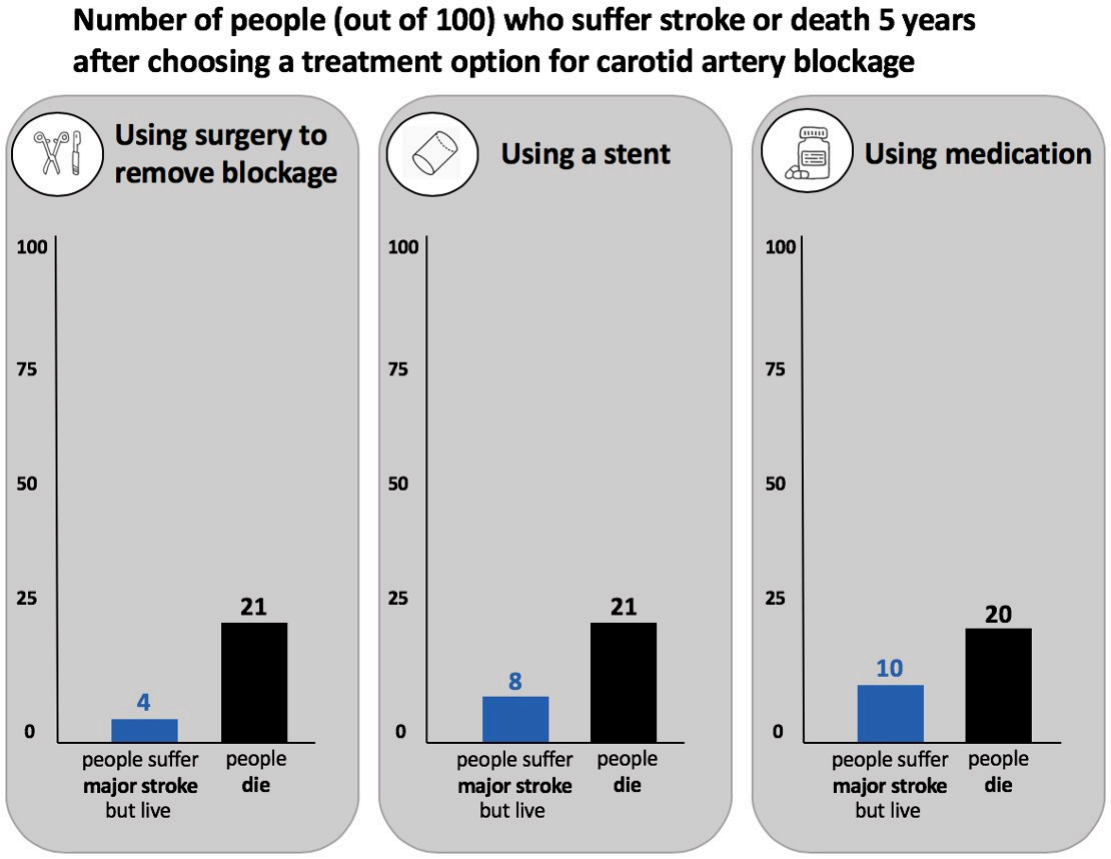
Bar graph presented in the survey.

We designed the bar graph (see Fig 3) based on feedback provided by ten people visiting a senior center in Lebanon, New Hampshire (see S2 Table for user-testing details). The feedback led us to modify the title of our bar graph to be: ‘number of people (out of 100) who suffer stroke or death 5 years after choosing a treatment option for carotid artery blockage’. We used the same treatment titles as for the icon array: ‘using surgery to remove blockage’, ‘using a stent’, and ‘using medication’. An icon (simple image of the relevant intervention) was also added to the title area. Below each title, were two vertical bars aligned to the y-axis scale of 0 to 100%. The length of each bar was proportional to the risk. The first bar, in blue, represented the number of ‘people who suffer major stroke but live’ and the second bar, in black, represented the number of people who died (‘people die’). We presented a number above each bar to indicate the risk associated with each outcome. Although similar to a generic bar chart, the meaningful difference is the addition of numbers, plain text and icons which are designed to enhance the format to help users better understand the magnitude of risk.

### Outcomes

The primary outcome measure was *understanding* based on three multiple-choice questions:

1. ‘Five years after the procedure, which treatment option has the *highest* chance of major stroke?’
2. ‘Five years after the procedure, which treatment option has the *lowest* chance of major stroke?’
3. ‘Which treatment option has the highest risk of major stroke and death five years after the procedure?’

Participants had to select the correct treatment option for all three questions to be categorized as having a high understanding of the risk of stroke and death for each carotid occlusion treatment option. We set this threshold prior to data collection due to the number of questions and because we wanted to impose a high standard to identify users with high understanding. Setting a threshold made it easier to categorize and differentiate participants with a high or low understanding of the risk information.

We collected data on the following outcomes:

1. Health literacy.
  Health literacy was evaluated using Chew’s three-item measure which contained the following questions: (i) How confident are you filling out medical forms by yourself?; (ii) How often do you have someone (like a family member, friend, hospital/clinic worker, or caregiver) help you read hospital materials?; and (iii) How often do you have problems learning about your medical condition because of difficulty understanding written information? [35, 36]. Patients who circled ‘extremely’ on the Likert-type scale for the first question, and ‘none of the time’ for the last two questions were categorized as having high health literacy skills.
2. Objective numeracy and graph literacy.
  Objective numeracy, defined as the ‘ability to understand numbers or percentages’ [22], is a predictor of an individual’s decision-making skills [37] and is directly associated with understanding risk information [38]. We used Schwartz’s validated, three-item numeracy measure [39]. The first item assessed basic familiarity with probability, the second asks respondents to convert a percentage (1%) to a proportion (10 in 1000), and the third item reverses this task, asking the respondent to convert a proportion (1 in 1000) to a percentage (0.1%) [23, 39].
  Graph literacy is the ‘ability to understand graphically-presented information’ [25]. We used Okan’s four-item scale to assess graph literacy [25], where participants: (i) are asked to estimate the percentage of people who died from three cancer types from a pie chart; (ii) view two line graphs and are asked to indicate which treatment leads to a larger decrease in the percentage of sick patients; (iii) view an icon array and are asked to indicate how many more men than women there were among 100 patients with disease X; and (iv) view two bar graphs and are asked to indicate which treatment led to a larger decrease in the percentage of patients who died (see S3 Table for details) [25]. Both graph literacy and objective numeracy scales used multiple choice formats. To be categorized as having high versus low objective numeracy and graph literacy proficiency, correct answers to all items were required. We opted to create categories to clearly differentiate participants with high and low objective numeracy and graph literacy proficiency. We made the decision prior to data collection to sum the scales to determine proficiency because we believe the two constructs—objective numeracy and graph literacy—are similar, and when combined, can provide a more complete assessment of the user’s literacy skills. We also wanted to set a high standard for identifying high proficiency users, and that is the reason why we set a perfect score (7/7) as the threshold for high proficiency.
3. Participants were asked to indicate the graphical display format that they preferred when considering the risk of major stroke or death for each carotid artery stenosis treatment option and asked to describe their reason for their preference in an open textbox.

### Procedure

Our survey had a total of 21 questions (see Table 1 for the survey structure). After reading information about the study, participants consented and proceeded to complete the survey. All participants completed questions about age, gender, race, educational attainment, income level, and health literacy, as well as the objective numeracy and graph literacy measures. Qualtrics ensured that an equal number of participants with high and low objective numeracy and graph literacy proficiency were present in each arm of the trail. The Qualtrics survey was configured to randomly assign participants to view one of the two graphical risk display formats (icon array or bar graph) and answer three questions to assess their understanding of the risk information presented. The last section presented versions of the two graphical display formats side-by-side, and participants were asked to select their preferred format and to explain *why* they preferred that format. Participants were not able to change responses once they completed a section in the survey. See S3 Table to view the survey.

**Table 1:** Overview of survey structure.

Page Number	Section	Number of questions
1	Introduction and consent	1
2	Demographics	5
3	Health literacy	3
4	Objective numeracy	3
5	Graph literacy	4
6	Understanding*	3
7	Preferred visual format	2

*User was randomized to view one graphical display format.

### Sample size calculation

Throughout both aims we used a p-value threshold of <0.05 to indicate statistical significance. We assumed that a clinically meaningful effect size equated to a 0.2 standard deviation change on the knowledge scale, a relatively small effect-size for studies on preferences of graphical display formats. In order to be able to reliably detect an effect of this size, we determined that we needed 394 participants. Here “reliably detect” translated to having a power of 0.80 to demonstrate a significant change between the groups using a two-sided test of significance 0.05-level test.

### Data collection and analysis

Data was collected until the sample size was attained. Survey responses were collected over a three-day period.

To address aim 1, we conducted a binary logistic regression analysis to examine possible associations between our dependent variable (0 = low understanding, 1 = high understanding) and the following independent categorical variables: age, gender, race, educational attainment, income level, health literacy, proficiency (0 = low numeracy and graph literacy, 1 = high numeracy and graph literacy), format (0 = icon array, 1 = bar graph) and graphical display format preference (0 = icon array, 1 = bar graph).

To address aim 2, we conducted a logistic regression using ‘proficiency’ (0 = low numeracy and graph literacy, 1 = high numeracy and graph literacy) as the dependent variable and the following independent categorical variables: age, gender, race, educational attainment, income level, health literacy, understanding (0 = low understanding, 1 = high understanding) and graphical display format preference (0 = icon array, 1 = bar graph). We tested for interaction effects by adding the products of the format variable and the three variables (education, annual income, health literacy) thought to interact with format to the model. We also conducted a sensitivity analysis by iteratively excluding participants based on their objective numeracy and graph literacy score (i.e., removing those who achieved 6/7 or 7/7 from the sample and conducting the analysis) to assess the effect on understanding scores and format preferences.

To address aim 3, two researchers (PS and DS) independently reviewed and coded each open-ended response. We tagged 30 responses using short-phrase labels or codes and then compared codebooks to ensure inter-rater agreement. We then proceeded to independently code the remaining responses using the agreed upon codebook, and then convened to group the codes into categories. Any coding disagreements were resolved by a third researcher (GE).

## Results

### Participant characteristics

A total of 523 participants consented to participate in the research study: 72 left the survey before completion, and 44 were excluded because they completed the survey in less than three minutes. Of the remaining 407 participants, 197 were randomly assigned to view the icon array, and 210 were assigned to view the bar graph. Overall, 60% (n = 244) of the participants were female, 80% (n = 327) were White, 60% (n = 244) had low health literacy, 43% (n = 173) had a bachelor’s degree, and 50% (n = 203) had low objective numeracy and graph literacy. There were no statistically significant baseline differences in the participants allocated to the two arms. See Table 2 for more details.

**Table 2:** Baseline measures.

	Icon Array	Bar graph	p-value*
	(n = 197)	(n = 210)	
**Age**			.86
18–24, n (%)	9 (5)	12 (6)	
25–34, n (%)	42 (21)	42 (20)	
35–44, n (%)	39 (20)	35 (17)	
45–54, n (%)	33 (17)	40 (19)	
55–64, n (%)	36 (18)	34 (16)	
65 or older, n (%)	38 (19)	47 (22)	
Prefer not to respond, n (%)	0 (0)	0 (0)	
**Gender**			.38
Male, n (%)	83 (42)	78 (37)	
Female, n (%)	113 (57)	131 (62)	
Other, n (%)	1 (1)	0 (0)	
Prefer not to respond, n (%)	0 (0)	1 (1)	
**Race**			.82
White, n (%)	159 (81)	168 (80)	
Black/African-American, n (%)	14 (7)	19 (9)	
Hispanic, n (%)	9 (5)	5 (2)	
Asian, n (%)	13 (7)	15 (7)	
American Indian, n (%)	1 (1)	1 (1)	
Other, n (%)	1 (1)	1 (1)	
Prefer not to respond, n (%)	0 (0)	1 (1)	
**Education**			.40
Completed some high school, n (%)	0 (0)	3 (1)	
High school graduate, n (%)	18 (9)	16 (8)	
Completed some college, n (%)	30 (15)	22 (11)	
Associate degree, n (%)	10 (5)	14 (7)	
Bachelor’s degree, n (%)	86 (44)	87 (41)	
Completed some postgraduate training, n (%)	8 (4)	14 (7)	
Master’s degree, n (%)	30 (15)	41 (20)	
PhD, MD, or JD, n (%)	12 (6)	9 (4)	
Other advanced degree beyond a master’s degree, n (%)	3 (2)	3 (1)	
Prefer not to respond, n (%)	0 (0)	1 (1)	
**Annual Income**			.79
<$25,000, n (%)	29 (15)	32 (15)	
$25,000-$34,999, n (%)	18 (9)	17 (8)	
$35,000-$49,999, n (%)	29 (15)	27 (13)	
$50,000-$74,999, n (%)	43 (22)	54 (26)	
$75,000-$99,999, n (%)	24 (12)	28 (13)	
$100,000-$149,000, n (%)	28 (14)	33 (16)	
$150,000 or more, n (%)	20 (10)	12 (6)	
Prefer not to respond, n (%)	6 (3)	7 (3)	
**Health literacy**			.14
Low, n (%)	124 (63)	120 (57)	
High, n (%)	73 (37)	90 (43)	
**Objective numeracy and graph literacy**			.36
Low^+^, n (%)	96 (49)	107 (51)	
High^+^, n (%)	101 (51)	103 (49)	
Objective numeracy 3/3 score, n (%)	119 (60)	125 (60)	
Graph literacy 4/4 score, n (%)	114 (58)	110 (52)	

*Based on chi-square tests.

^+^High = correct answers on all items; Low = one or more incorrect answers.

### Relationship between graphical display format and understanding

**Fig 4:**
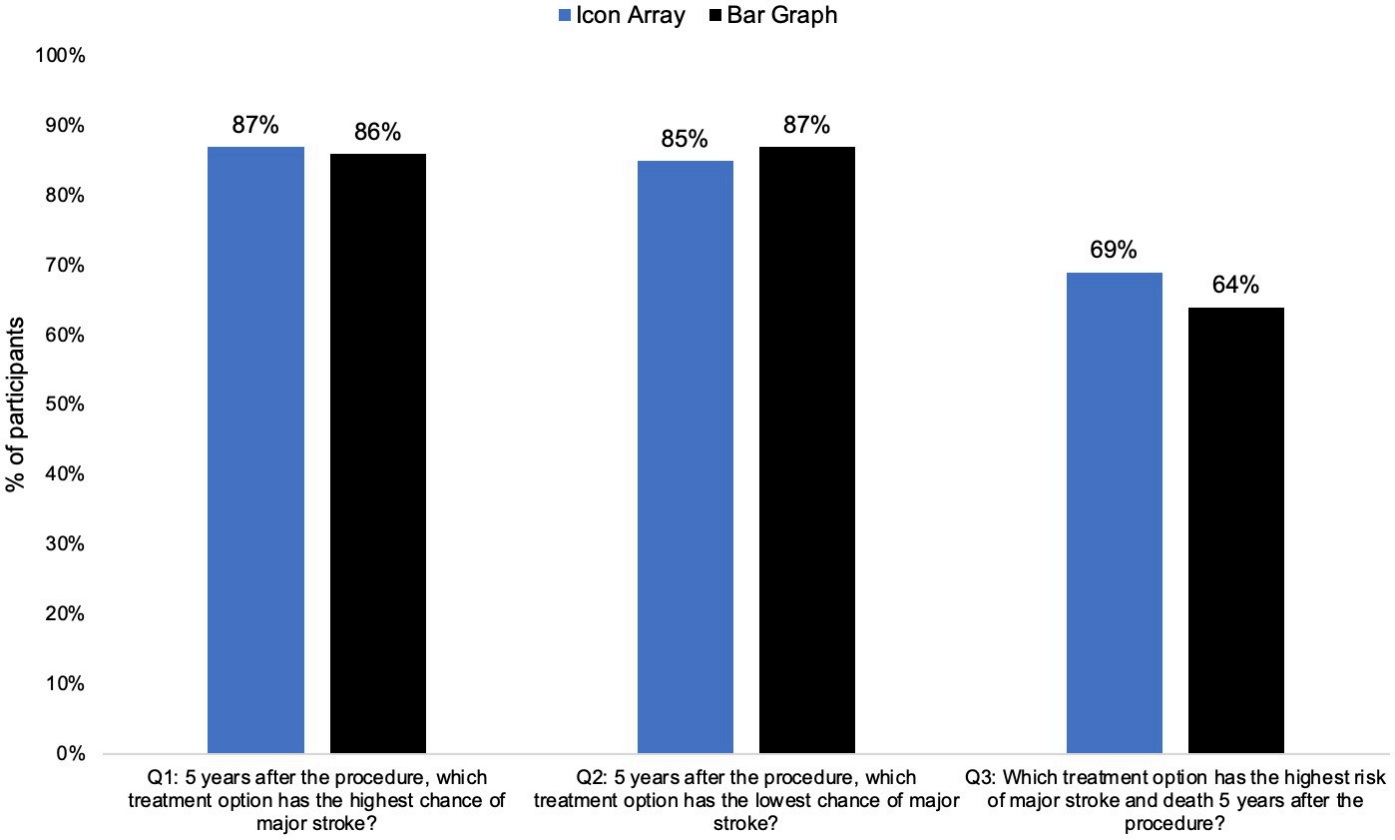
The percentage of participants in each trial arm that correctly answered each knowledge question in the survey.

Understanding the risk of stroke and death associated with each carotid artery stenosis treatment option did not differ significantly between those randomized to the bar graph compared to the icon array: 61% (n = 121/197) in the icon array arm had high understanding, and 59% (n = 123/210) of participants in the bar graph arm had high understanding of the risk information. Responses to the first two questions that assessed understanding did not differ significantly between those randomized to the bar graph compared to the icon array: 80% (n = 157/197) in the icon array, and 82% (n = 172/210) of participants in the bar graph arm had a high understanding. Responses to the last question assessing risk understanding also did not differ significantly between the two arms: 69% (n = 136/197) in the icon array arm, and 64% (n = 135/210) in the bar graph arm had high understanding. See Fig 4 to compare the percentage of participants in each trial arm that correctly answered each knowledge question in the survey.

The regression using ‘understanding’ as the dependent variable showed that high understanding was associated with high objective numeracy and graph literacy (p<0.01) and preference for the bar graph (p<0.01). See S4 Table for details.

### Analysis with high and low objective numeracy and graph literacy subgroups

The regression using ‘proficiency’ (0 = low numeracy and graph literacy, 1 = high numeracy and graph literacy) as the dependent variable showed that participants with high objective numeracy and graph literacy were associated with high understanding (p<0.01) and a preference for the bar graph format (p = 0.01). Of the interaction terms tested, none were found to be statistically relevant. See S5 Table for details. Our sensitivity analysis also did not yield significant changes to understanding of risk information or preferences. This means that understanding and preferences remained the same for each format despite lowering the threshold of numeracy and graph literacy proficiency.

Only 19% (n = 38/197) of participants allocated to the icon array with low objective numeracy and graph literacy had high understanding. In comparison, of participants with low objective numeracy and graph literacy allocated to the bar graph arm, 23% (n = 48/210) had high understanding. See Table 3 for details.

**Table 3:** Comparison of participants’ understanding and objective numeracy and graph literacy proficiency for each trial arm.

	Icon Array			Bar graph		
	n = 197			n = 210		
	Low understanding	High understanding	Preference for Icon Array	Low understanding	High understanding	Preference for Bar graph
Low objective numeracy and graph literacy, n (%)	58 (29)	38 (19)	28 (14)	59 (28)	48 (23)	78 (37)
High objective numeracy and graph literacy, n (%)	18 (9)	83 (42)	36 (18)	28 (13)	75 (36)	57 (27)

### Graphical display format preference

The bar graph format was preferred by 66% (n = 268/407) of the participants. Of those who preferred the bar graph, 88% (n = 236/268) stated it was easier to read and understand the risk data compared to the icon array. A participant captured the gist of why the majority preferred the bar graph:

*I find that showing all the little people icons in the icon array is somewhat redundant and busy looking. Showing me a graph that simply states the number of deaths, and the number of strokes is much more straightforward*.

(Participant 195; high objective numeracy and graph literacy)

Participants said that the number sitting above each bar was the key feature that helped compare the risks of stroke and death for each treatment option:

*For me*, *the two bars with the numbers on top of them were much easier to understand immediately and there was no need for a key*.

(Participant 249; high objective numeracy and graph literacy)

*The bar graph is easier to read … The icon array also shows the numbers, but it is a bit more confusing to look at*.

(Participant 264; high objective numeracy and graph literacy)

The icon array format was preferred by 34% (n = 139/407) of the participants. Of those who preferred the icon array, 47% (n = 66/139) of participants said it was because the format was visually appealing. Participants felt that the color-coding helped them differentiate between the probability of no event and the risk of stroke and death:

*The colors assist as the individual icons take up the same amount of space. It is easier to see how many live, have a stroke or die*.

(Participant 285; high objective numeracy and graph literacy)

Also, of those who preferred the icon array, 33% (n = 46/139) stated that the icon array was more informative compared to the bar graph due to the presentation of the probability of no event. This helped participants understand that the majority of patients did not suffer stroke or death five years after an intervention to treat carotid artery stenosis:

*Icon Array—because it provides a visual representation of the number of people out of 100 that had neither stroke nor death occur, which is something not depicted in the bar graph*.

(Participant 404; high objective numeracy and graph literacy)

Table 4 compares the reasons why participants preferred one format over the other.

**Table 4:** Rationale provided by users as to why they prefer the bar graph or the icon array.

Reasons for icon array preference (n = 139/407)	Reasons for bar graph preference (n = 268/407)
Visually appealing (66/139; 47%)	Easier to understand and read (236/268; 88%)
More informative (46/139; 33%)	Less complicated (n = 20/268; 7%)
Easier to understand (27/139; 19%)	Increased clarity (n = 9/268; 3%)
	Easier to do math (n = 2/268; 1%)
	More trustworthy (n = 1/268; 0%)

## Discussion

### Summary of main findings

Understanding the risk of stroke and death associated with each carotid artery stenosis treatment option did not differ significantly between those randomized to the bar graph compared to the icon array, irrespective of objective numeracy and graph literacy proficiency. Understanding was associated with high objective numeracy and graph literacy proficiency and preference for the bar graph. The majority preferred viewing the risk information using the bar graph format. Those who preferred the bar graph stated it was easier to read and differentiate the risk of stroke and death due to the numbers sitting atop each bar. The minority who preferred the icon array cited the color-coding scheme which helped visualize the different probabilities, including that of ‘no event’ which they felt was absent in the bar graph.

### Strengths and limitations

Our study contains several strengths: we user-tested each graphical display format with users across the health literacy spectrum, included an even number of participants with high and low objective numeracy and graph literacy proficiency based on validated measures, randomized the graphical display formats in a concealed fashion, assessed understanding of risk, and provided the opportunity to describe *why* participants preferred a particular format.

Our study does contain some limitations. The survey was administered online, so the demographics of our sample, which was mostly female, White and well-educated, is not reflective of the general population. Approximately 43% of participants in our sample had a bachelor’s degree which is much higher in comparison to the 21% in the general US population who have a bachelor’s degree (based on 2017 data) [40]. We acknowledge omission of the participant’s geographic location; however, we believe that the demographic variables we collected (age, gender, race, education, annual income, health literacy) provide a comprehensive description of our participants. Second, we did not seek information on the participants’ health condition, but we can assume that few, if any, participants were actually facing the decision to treat carotid artery stenosis. Considering the evidence that emotions often supersede rationality when cognitively processing risk information [6], it is unclear if our results would have been different with a sample that consisted of participants needing to make a real-life decision on how to treat carotid artery stenosis. The absence of a control group can also be considered a limitation, however we felt that the randomized design enabled us to draw meaningful conclusions on user knowledge and preference when comparing the bar graph to the icon array. Also, the two formats used in this study were independently refined after iterations of interviews at different time points. As indicated in the Methods, the icon array was user-tested as part of a separate qualitative study [31]. Due to the user-testing of each format occurring with different samples, the titles of the two formats differ. In addition, the bar graph does not present the probability of ‘no event’. These differences are a limitation, but they are also a result of feedback from the user-testing phase of development. Further, a participant’s preference for a type of graphical display format does not necessarily translate to having a greater understanding of the risk information, so the findings regarding preference need to be interpreted with caution [41]. Lastly, we are cognizant that the formats used in this study focus on one particular health condition and the three related options to treat that condition. The impact of different graphical display formats on understanding is context dependent.

### Results in context

Despite their popularity, a paucity of data exists to demonstrate the superiority of icon arrays over other graphical display formats to improve risk understanding among those with lower objective numeracy and lower graph literacy. A recent cross-sectional, online survey study by Durand et al. found that Medicaid enrollees had significantly higher risk comprehension scores when using bar graphs compared to icon arrays [42]. The sample also preferred viewing the risk data in a bar graph format instead of the icon array. Preference for viewing risk information using bar graphs also aligns with previous research that shows that presenting bars and scales vertically is associated with processing information with greater speed and accuracy, and decreases framing effects [12, 15, 19]. However, it is important to note that some participants in our sample preferred the icon array because it presented the ‘whole picture’.^8^ Evidence indicates that only presenting the numerator leads to an inflated risk perception, so the probability of ‘no event’ was important for some participants [2, 8, 18].

### Future research

Future studies should compare the bar graph to alternative graphical display formats like pie charts or line graphs to determine its efficacy for improving risk comprehension, particularly for those with lower literacy skills. We need to identify the elements of the bar graph that impact understanding of risk information, and if it is preferred over other formats across different patient populations and decisional contexts. More broadly, our study highlights the need to find better ways to convey two or more risk variables simultaneously (i.e., risk of stroke and death) or risk magnitudes that are less obvious. Displaying the risk differences (however small) so they are salient will also help patients understand risks that change in a non-equivalent way with time [43]. This is critical considering that patients may have different preferences over different time horizons when shown the comparative risk of a negative event in the near versus the longer-term future. Future work should also aim to reach low literate groups in community settings and use qualitative methods (i.e., think aloud technique) to identify formats that improve understanding of risk information.

## Conclusion

Understanding the risk of stroke and death associated with each carotid artery stenosis treatment option did not differ significantly between those randomized to the bar graph compared to the icon array, irrespective of objective numeracy and graph literacy proficiency. A high level of risk understanding was associated with high objective numeracy and graph literacy proficiency, and a preference for the bar graph format. Overall, the majority participants preferred viewing the risk information using the bar graph as opposed to the icon array.

## Supporting information

S1 Table CONSORT checklist for reporting results of online surveys. (PDF)

S2 Table The two bar graph prototypes that were tested with low health literacy community members. (PDF)

S3 Table The participant survey. (PDF)

S4 Table Logistic regression analysis to address the first study aim. (PDF)

S5 Table Logistic regression analysis to address the second study aim. (PDF)
