# Supplementary material for: Comparing the impact of an icon array versus a bar graph on preference and understanding of risk information: Results from an online, randomized study
Source: PLoS One. 2021 Jul 23;16(7):e0253644. doi: 10.1371/journal.pone.0253644 (PMC8301663; doi:10.1371/journal.pone.0253644)
Supplement: S1 Table — (PDF) [file pone.0253644.s001.pdf]

Section/Topic	Item no.	Checklist Item	Page #
**Title and abstract**			
	1a	Identification as a randomised trial in the title	1
	1b	Structured summary of trial design, methods, results, and conclusions	2
**Introduction**			
Background and	2a	Scientific background and explanation of rationale	3
Objectives	2b	Specific objectives or hypotheses	4
**Methods**			
Trial design	3a	Description of trial design (such as parallel, factorial) including allocation ratio	4
	3b	Important changes to methods after trial commencement (such as eligibility criteria), with reasons	N/A
Participants	4a	Eligibility criteria for participants	4
	4b	Settings and locations where the data were collected	4
Interventions	5	The interventions for each group with sufficient details to allow replication, including how and when they were actually administered	5

Outcomes	6a	Completely defined pre-specified primary and secondary outcome measures, including how and when they were assessed	5-7
	6b	Any changes to trial outcomes after the trial commenced, with reasons	N/A
Sample size	7a	How sample size was determined	7
	7b	When applicable, explanation of any interim analyses and stopping guidelines	N/A
Randomization (sequence generation)	8a	Method used to generate the random allocation sequence	7
	8b	Type of randomisation; details of any restriction (such as blocking and block size)	N/A
Allocation (concealment mechanism)	9	Mechanism used to implement the random allocation sequence (such as sequentially numbered containers), describing any steps taken to conceal the sequence until interventions were assigned	7
Implementation	10	Who generated the random allocation sequence, who enrolled participants, and who assigned participants to interventions	7
Blinding	11a	If done, who was blinded after assignment to interventions (for example, participants, care providers, those assessing outcomes) and how	7
	11b	If relevant, description of the similarity of interventions	5

Statistical methods	12a	Statistical methods used to compare groups for primary and secondary outcomes	7-8
	12b	Methods for additional analyses, such as subgroup analyses and adjusted analyses	7-8
**Results**			
Participant flow (a diagram is strongly recommended)	13a	For each group, the numbers of participants who were randomly assigned, received intended treatment, and were analysed for the primary outcome	8
	13b	For each group, losses and exclusions after randomisation, together with reasons	8
Recruitment	14a	Dates defining the periods of recruitment and follow-up	7
	14b	Why the trial ended or was stopped	N/A
Baseline data	15	A table showing baseline demographic and clinical characteristics for each group	8
Numbers analyzed	16	For each group, number of participants (denominator) included in each analysis and whether the analysis was by original assigned groups	8
Outcomes and estimation	17a	For each primary and secondary outcome, results for each group, and the estimated effect size and its precision (such as 95% confidence interval)	8-10
	17b	For binary outcomes, presentation of both absolute and relative effect sizes is recommended	8-10

Ancillary analyses	18	Results of any other analyses performed, including subgroup analyses and adjusted analyses, distinguishing pre-specified from exploratory	8-9
Harms	19	All important harms or unintended effects in each group	N/A
**Discussion**			
Limitations	20	Trial limitations, addressing sources of potential bias, imprecision, and, if relevant, multiplicity of analyses	10-11
Generalizability	21	Generalisability (external validity, applicability) of the trial findings	11
Interpretation	22	Interpretation consistent with results, balancing benefits and harms, and considering other relevant evidence	11-12
**Other information**			
Registration	23	Registration number and name of trial registry	N/A
Protocol	24	Where the full trial protocol can be accessed, if available	N/A
Funding	25	Sources of funding and other support (such as supply of drugs), role of funders	13
