# Supplementary material for: Comparing the impact of an icon array versus a bar graph on preference and understanding of risk information: Results from an online, randomized study
Source: PLoS One. 2021 Jul 23;16(7):e0253644. doi: 10.1371/journal.pone.0253644 (PMC8301663; doi:10.1371/journal.pone.0253644)
Supplement: S2 Table — (PDF) [file pone.0253644.s002.pdf]

Two bar graph prototypes were developed with the guidance of two visual design experts (GN and JF). The prototypes were presented to ten persons who frequent the Upper Valley Senior Center in Lebanon, New Hampshire. Participants were 55 years of age or older, mostly female (n=8) with low objective numeracy skills (n=7), and half had an annual income of $25,000 or less (see table below the prototypes for complete sample demographics). They completed informed consent, a short demographic questionnaire, and used their preferred prototype to answer three knowledge questions. Cognitive interviews were then conducted to assess their understanding of the risk data presented, elements of the format that needed refining, and their preferred prototype. A $10 gas card was given to each participant to compensate them for their time.

**Characteristic**	**N**
Age	
65 or older	7
55-64	3
45-54	0
35-44	0
25-34	0
18-24	0
Gender	
Male	2
Female	8
Race	
White	10

Black/African-American	0
Asian	0
Hispanic	0
Native Hawaiian/Other Pacific Islander	0
American Indian	0
Alaska Native	0
Other	0
Education	
Completed some high school	1
High school graduate	1
Completed some college	2
Associate degree	1
Bachelor’s degree	5
Completed some postgraduate	0
Master’s degree	0
PhD, MD, or JD	0
Other advanced degree beyond a master’s degree	0
Income	
<$25k	5

$25k-$35k	1
$35k-$50k	2
$50k-$75k	1
$75k-$99k	1
$100k-$150k	0
$150k<	0
Health Literacy	
High	5
Low	5
Objective Numeracy	
High	3
Low	7
Knowledge (3 questions)	
Perfect knowledge score	5
One or more knowledge question error	5
