# Supplementary material for: Comparing the impact of an icon array versus a bar graph on preference and understanding of risk information: Results from an online, randomized study
Source: PLoS One. 2021 Jul 23;16(7):e0253644. doi: 10.1371/journal.pone.0253644 (PMC8301663; doi:10.1371/journal.pone.0253644)
Supplement: S3 Table — (PDF) [file pone.0253644.s003.pdf]

Demographic section

Please select age category:

- 18-24 years
- 25-34 years
- 35-44 years
- 45-54 years
- 55-64 years
- 65 or older
- Prefer not to respond

Please select your gender:

- Male
- Female
- Other (Free text response box was provided)
- Prefer not to respond

Please select your race:

- Black/African American
- Asian
- Hispanic
- Native Hawaiian/Other Pacific Islander
- White
- American Indian
- Alaska Native
- Other (Free text response box was provided)
- Prefer not to respond

Please select your highest level of education:

- Completed some high school
- High school graduate
- Completed some college
- Associate degree
- Bachelor's degree
- Completed some postgraduate
- Master's degree
- PhD, MD, or JD
- Other advanced degree beyond a Master's degree
- Prefer not to respond

Please select your annual income level:
- Less than $25,000
- $25,000 to $34,999
- $35,000 to $49,999
- $50,000 to $74,999
- $75,000 to $99,999
- $100,000 to $149,999
- $150,000 or more
- Prefer not to respond

Health literacy section

How confident are you filling out medical forms by yourself?
- Extremely
- Quite a bit
- Somewhat
- A little bit
- Not at all

How often do you have someone (like a family member, friend, hospital/clinic worker, or caregiver) help you read hospital materials?
- Always
- Often
- Sometimes
- Occasionally
- Never

How often do you have problems learning about your medical condition because of difficulty understanding written information?
- Always
- Often
- Sometimes
- Occasionally
- Never

Objective numeracy section

1) "Imagine that we flip a fair coin 1,000 times. What is your best guess about how many times the coin would come up heads in 1,000 flips? ____times out of 1,000."
- 25
- 50

- 250
- 500

2) “In the BIG BUCKS LOTTERY, the chance of winning a $10 prize is 1%. What is your best guess about how many people would win a $10 prize if 1000 people each buy a single ticket to BIG BUCKS?____person(s) out of 1,000.”

- 1 person
- 2 people
- 10 people
- 100 people

3) “In ACME PUBLISHING SWEEPSTAKES, the chance of winning a car is 1 in 1,000. What percent of tickets to ACME PUBLISHING SWEEPSTAKES win a car?____%.”

- 0.01%
- 0.1%
- 1%
- 10%

Graph literacy section

1)
Approximately what percentage of people who die from cancer die from colon cancer, breast cancer, and prostate cancer taken together? ___ %

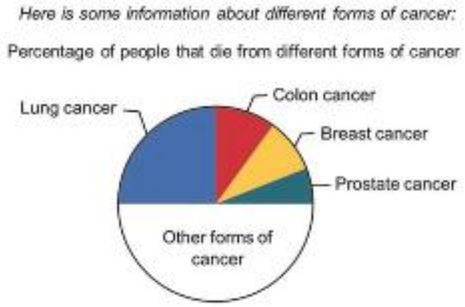

2)

In a magazine you see two advertisements, one on page 5 and another on page 12. Each is for a different drug for treating heart disease, and each includes a graph showing the effectiveness of the drug compared to a placebo (sugar pill).

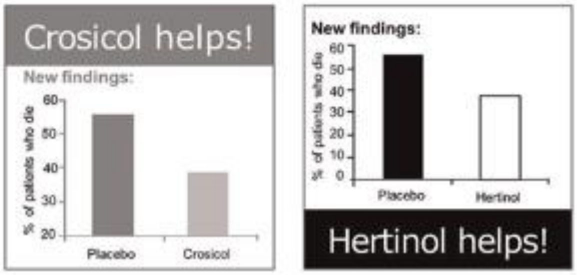

Compared to the placebo, which treatment leads to a larger decrease in the percentage of patients who die?

- Crosicol
- Hertinol
- They are equal
- Can’t say

3)

The following figure shows the number of men and women among patients with disease X. The total number of circles is 100.

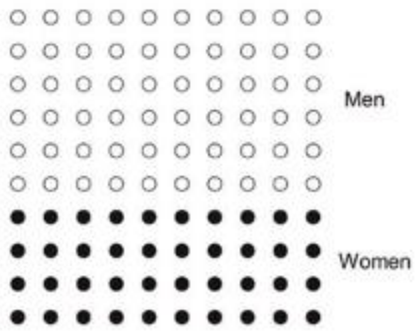

How many more men than women are there among 100 patients with disease X? ___ Men

4)

In the newspaper you see two advertisements, one on page 15 and another on page 17. Each is for a different treatment of psoriasis, and each includes a graph showing the effectiveness of the treatment over time.

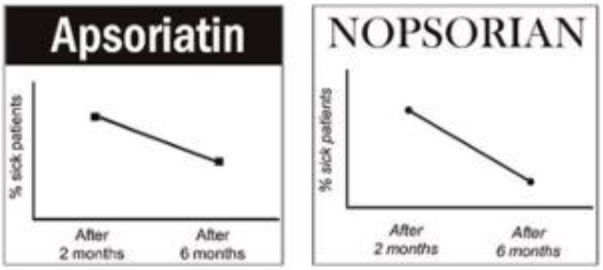

Which of the treatments contributes to a larger decrease in the percentage of sick patients?

- Apsoriatin
- Nopsorian
- They are equal
- Can’t say

[**Randomization to a graphical display format**]

Five years after the procedure, which treatment option has the highest chance of major stroke?

- Using surgery to remove blockage
- Using a stent
- Using medication

Five years after the procedure, which treatment option has the lowest chance of major stroke?

- Using surgery to remove blockage
- Using a stent
- Using medication

Which treatment option has the highest risk of major stroke and death five years after the procedure?

- Using surgery to remove blockage
- Using a stent
- Using medication

[Both graphical display formats presented side-by-side]

From the two options above, which do you prefer to better understand risk information?

- Icon Array
- Bar graph

Please describe why you prefer your selection in as much detail as possible.
