# Supplementary material for: Comparing the impact of an icon array versus a bar graph on preference and understanding of risk information: Results from an online, randomized study
Source: PLoS One. 2021 Jul 23;16(7):e0253644. doi: 10.1371/journal.pone.0253644 (PMC8301663; doi:10.1371/journal.pone.0253644)
Supplement: S4 Table — (PDF) [file pone.0253644.s004.pdf]

**Variable**	**B**	**S.E.**	**Wald**	**df**	**Sig.**	**OR**	**95% CI**
**Age**							
18-24	-	-	-	-	-	-	-
25-34	-0.20	0.58	0.11	1	0.74	0.83	0.27-2.57
35-44	0.55	0.38	2.18	1	0.14	1.74	0.83-3.63
45-54	-0.29	0.39	0.55	1	0.46	0.75	0.35-1.60
55-64	0.32	0.38	0.70	1	0.40	1.38	0.65-2.91
65<	0.63	0.39	2.62	1	0.11	1.87	0.88-4.01
Prefer not to respond	-	-	-	-	-	-	-
**Gender**							
Male	-	-	-	-	-	-	-
Female	19.66	51514	0.00	1	1.00	-	-
Other	19.71	51514	0.00	1	1.00	-	-
Prefer not to respond	40.83	65339	0.00	1	1.00	-	-
**Race**							
White	-	-	-	-	-	-	-

Black/African-American	-19.61	27816	0.00	1	1.00	-	-
Hispanic	-20.13	27816	0.00	1	1.00	-	-
Asian	-20.10	27816	0.00	1	1.00	-	-
American Indian	-18.78	27816	0.00	1	1.00	-	-
Other	-40.64	38090	0.00	1	1.00	-	-
Prefer not to respond	-	-	-	-	-	-	-
**Education**							
Completed some high school	-	-	-	-	-	-	-
High school graduate	-20.42	16256	0.00	1	1.00	-	-
Completed some college	-20.36	16256	0.00	1	1.00	-	-
Associate degree	-20.00	16256	0.00	1	1.00	-	-
Bachelor’s degree	-19.86	16256	0.00	1	1.00	-	-
Completed some postgraduate training	-19.96	16256	0.00	1	1.00	-	-
Master’s degree	-19.93	16256	0.00	1	1.00	-	-
PhD, MD, or JD	-20.23	16256	0.00	1	1.00	-	-
Other advanced degree beyond a master’s degree	-20.34	16256	0.00	1	1.00	-	-

Prefer not to respond	-	-	-	-	-	-	-
**Annual Income**							
<$25,000	-	-	-	-	-	-	-
$25,000-$34,999	-1.15	0.84	1.88	1	0.17	0.32	0.06-1.64
$35,000-$49,999	-1.15	0.88	1.73	1	0.19	0.32	0.06-1.76
$50,000-$74,999	-1.06	0.84	1.62	1	0.20	0.35	0.07-1.77
$75,000-$99,999	-0.93	0.82	1.29	1	0.26	0.40	0.08-1.97
$100,000-$149,000	-0.54	0.86	0.40	1	0.53	0.58	0.11-3.10
$150,000 or more	-0.85	0.84	1.03	1	0.31	0.43	0.08-2.20
Prefer not to respond	-1.17	0.90	1.72	1	0.19	0.31	0.05-1.79
**Health literacy**							
Low	-	-	-	-	-	-	-
High	-0.21	0.25	0.71	1	0.40	0.81	0.50-1.32
**Objective numeracy & graph literacy**							

Low	-	-	-	-	-	-	-
High	-1.49	0.29	27.38	1	0.00	0.23	0.13-0.39
**Format**							
Icon Array	-	-	-	-	-	-	-
Bar graph	0.10	0.23	0.18	1	0.67	1.11	0.70-1.75
**Preference**							
Icon array	-	-	-	-	-	-	-
Bar graph	-0.75	0.26	8.58	1	0.00	0.47	0.29-0.78
