# Supplementary material for: Comparing the impact of an icon array versus a bar graph on preference and understanding of risk information: Results from an online, randomized study
Source: PLoS One. 2021 Jul 23;16(7):e0253644. doi: 10.1371/journal.pone.0253644 (PMC8301663; doi:10.1371/journal.pone.0253644)
Supplement: S5 Table — (PDF) [file pone.0253644.s005.pdf]

Variable	B	S.E.	Wald	df	Sig.	OR	95% CI
**Age**							
18-24	-	-	-	-	-	-	-
25-34	0.85	0.83	1.04	1	0.31	2.34	0.46-12.0
35-44	-0.23	0.43	0.28	1	0.60	0.80	0.34-1.86
45-54	-0.26	0.45	0.33	1	0.57	0.77	0.32-1.87
55-64	-0.06	0.45	0.02	1	0.89	0.94	0.39-2.29
65<	0.22	0.48	0.21	1	0.65	1.25	0.49-3.18
Prefer not to respond	-	-	-	-	-	-	-
**Gender**							
Male	-	-	-	-	-	-	-
Female	19.84	51483	0.00	1	1.00	4.14E8	-
Other	19.74	51483	0.00	1	1.00	3.75E8	-
Prefer not to respond	39.23	65315	0.00	1	1.00	1.1E17	-
**Race**							
White	-	-	-	-	-	-	-
Black/African-American	-20.3	27911	0.00	1	1.00	0.00	-
Hispanic	-21.3	27911	0.00	1	1.00	0.00	-

Asian	-21.9	27911	0.00	1	1.00	0.00	-
American Indian	-20.0	27911	0.00	1	1.00	0.00	-
Other	-18.5	27911	0.00	1	1.00	0.00	-
Prefer not to respond	-	-	-	-	-	-	-
**Education**							
Completed some high school	-	-	-	-	-	-	-
High school graduate	-41.5	27490	0.00	1	1.00	0.00	-
Completed some college	-23.3	16007	0.00	1	1.00	0.00	-
Associate degree	-23.1	16007	0.00	1	1.00	0.00	-
Bachelor’s degree	-41.7	17724	0.00	1	1.00	0.00	-
Completed some postgraduate training	-19.7	16007	0.00	1	1.00	0.00	-
Master’s degree	-19.7	16007	0.00	1	1.00	0.00	-
PhD, MD, or JD	-19.4	16007	0.00	1	1.00	0.00	-
Other advanced degree beyond a master’s degree	-19.2	16007	0.00	1	1.00	0.00	-
Prefer not to respond	-	-	-	-	-	-	-
**Annual Income**							
<$25,000	-	-	-	-	-	-	-

$25,000-$34,999	0.81	1.06	0.59	1	0.44	2.26	0.28-17.95
$35,000-$49,999	1.97	1.15	2.95	1	0.09	7.18	0.76-68.14
$50,000-$74,999	0.24	0.94	0.07	1	0.80	1.27	0.20-8.10
$75,000-$99,999	0.33	0.89	0.14	1	0.71	1.39	0.24-8.00
$100,000-$149,000	0.09	0.99	0.01	1	0.92	1.09	0.19-6.34
$150,000 or more	0.55	0.88	0.39	1	0.53	1.73	0.31-9.62
Prefer not to respond	1.24	0.95	1.70	1	0.19	3.44	0.54-22.06
**Health literacy**							
Low	-	-	-	-	-	-	-
High	-0.57	0.45	1.58	1	0.21	0.57	0.23-1.37
**Knowledge score**							
Low	-	-	-	-	-	-	-
High	-1.48	0.30	24.91	1	**0.00**	0.23	0.13-0.41
**Preference**							
Icon array	-	-	-	-	-	-	-
Bar graph	0.73	0.31	5.65	1	**0.02**	2.07	1.14-3.77
**Randomization**							

Icon Array	-	-	-	-	-	-	-
Bar graph	0.22	1.07	0.04	1	0.84	1.24	0.15-10.11
**Format*Education**	0.03	0.23	0.01	1	0.91	1.03	0.65-1.61
**Format* Annual income**	-0.00	0.16	0.00	1	0.99	1.00	0.73-1.61
**Format*Health literacy**	-0.43	0.59	0.53	1	0.47	0.65	0.20-2.08
